# DNA-Directed Assembly of Nanogold Dimers: A Unique Dynamic Light Scattering Sensing Probe for Transcription Factor Detection

**DOI:** 10.1038/srep18293

**Published:** 2015-12-18

**Authors:** Nianjia Seow, Yen Nee Tan, Lin-Yue Lanry Yung, Xiaodi Su

**Affiliations:** 1 Department of Chemical and Biomolecular Engineering, Faculty of Engineering, National University of Singapore, Singapore 119260, Singapore; 2 Institute of Material Research and Engineering, ASTAR (Agency for Science, Technology and Research), 3 Research Link, Singapore 117602

## Abstract

We have developed a unique DNA-assembled gold nanoparticles (AuNPs) dimer for dynamic light scattering (DLS) sensing of transcription factors, exemplified by estrogen receptor (ER) that binds specifically to a double-stranded (ds) DNA sequence containing estrogen response element (ERE). Here, ERE sequence is incorporated into the DNA linkers to bridge the AuNPs dimer for ER binding. Coupled with DLS, this AuNP dimer-based DLS detection system gave distinct readout of a single ‘complex peak’ in the presence of the target molecule (i.e., ER). This unique signature marked the first time that such nanostructures can be used to study transcription factor-DNA interactions, which DLS alone cannot do. This was also unlike previously reported AuNP-DLS assays that gave random and broad distribution of particles size upon target binding. In addition, the ERE-containing AuNP dimers could also suppress the light-scattering signal from the unbound proteins and other interfering factors (e.g., buffer background), and has potential for sensitive detection of target proteins in complex biological samples such as cell lysates. In short, the as-developed AuNP dimer probe coupled with DLS is a simple (mix and test), rapid (readout in ~5 min) and sensitive (low nM levels of ER) platform to detect sequence-specific protein-DNA binding event.

The investigation of important biomolecular events such as DNA mutation and gene transcription have been made possible with the advent of nanotechnology. Various nanosensing probes, such as metal nanoparticles, quantum dots, and silicon nanowires have been utilized to lend insight into the intertwining complexities between biomolecules, by transducing ‘invisible’ biological signals into measurable output^1^,^2^,^3^,^4^. In particular, gold nanoparticles (AuNPs) have added a new dimension to the realm of biosensing through their exhibition of localized surface plasmon resonance (LSPR)^5^. The strong absorbance and scattering characteristics of AuNPs at the visible light region render them as ideal sensing probes for various bioassay developments based on different optical responses. For example, the interparticle-distance dependent plasmonic coupling of AuNPs has been utilized to design colorimetric assays for biomolecular detection^6^,^7^. An example of AuNP-based colorimetric assays used in the study of gene-protein interaction is shown in the work of Ou and co-workers in which AuNPs containing complementary single strand DNA (ssDNA) are hybridized into assemblies as dsDNA are formed, in the process generating binding sites for the TATA binding protein. The DNA-protein complex protected the AuNP-assemblies from exonuclease III cleavage, which in turn allowed detection of the TATA-binding protein^8^. In another work by Fang and co-workers, *lac* operator-coated AuNPs aggregate when they are bridged by a common *lac* repressor protein. *Lac* repressor contains two DNA-binding domains, which allows concurrent binding to two AuNPs, inducing AuNP aggregation^9^. However, a high concentration of targets are needed in the colorimetric assay to aggregate the AuNP for appreciable color changes to be visibly perceived, which results in less than ideal sensitivity^10^,^11^. In addition, it is not suitable for use in coloured samples such as blood, which would interfere with the red to purple/blue transition in observations by the naked eye or using UV-*vis* spectroscopy.

Given that low sensitivity is one of the main limitations of gold nanoparticles-based colorimetric assays, methods such as biobarcode and silver staining amplifications have been carried out to address the problem, but it is still complicated and time consuming^12^,^13^. As such, a clever adaptation of DLS, typically used in nanoparticle characterization, for the detection of AuNPs and biomolecular diagnostics was first reported by Huo and co-workers. In successive works, they have shown that AuNP probes carrying recognition sequences for protein-protein binding and DNA-DNA hybridization would cluster and aggregate in the presence of their target binders. The increase in particle size led to a wholesale shift in the population distribution from tens of nm to the hundreds nm range^10^,^14^. Given that larger AuNPs show greater scattering cross section, the overall size increase from the aggregation amplifies the readout, further enhancing the sensitivity and clarity of the readout^15^,^16^,^17^. DLS can also detect 100 nm AuNPs at as low as fM level without added processing or amplifications, which makes DLS a sensitive platform for detecting AuNP-transduced biorecognition signals, and also diagnostic strategies involving increase in AuNP size. However, as in most aggregation-based systems, particle aggregation is an uncontrolled process, with the biomolecular targets causing the AuNPs to aggregate and grow extensively, leading to large variations in AuNP aggregate size and complex DLS readouts that are complicated to analyze especially for more subtle size changes^18^. Greater control over the probe-analyte interaction process is necessary to leverage the size growth of AuNPs, detected by DLS machine.

In this study, we report a novel design of well-defined Au dimeric nanostructures (AuNP dimers), coupled with DLS as a new sensing platform applied for studying sequence-specific binding interactions of estrogen receptor (ER) with its consensus DNA containing estrogen response element (ERE)^19^. ER is a protein biomarker that has significant implication in breast cancer prognosis and treatment^20^,^21^. A sensitive and selective method for its detection in a fast and simple manner is highly desirable. In our assay design, ERE is localized within the DNA linker used to bridge the two AuNPs to form the dimers. Unlike the previously reported DLS-AuNPs assays for cancer protein biomarkers (e.g. carcinoembryonic antigen) whose definitive readout was the increasing polydispersity and broadening size distribution of the antibody-tagged AuNP probe upon biomarker binding^22^,^23^, this AuNP dimer based DLS detection system showed distinct readout of a single ‘complex peak’ in the presence of the target molecule (i.e., ER). This unique optical signature marked the first time that such unique DNA-linked AuNP dimers can be used in conjunction with DLS platform to study sequence-specific transcription factor-DNA interactions.

## Results

### Design of DNA-directed assembly of AuNP dimer for ER (β subtype) detection

**Figure 1:**
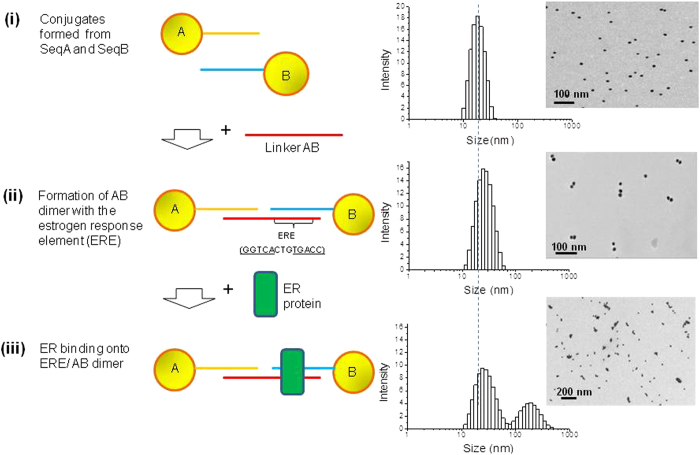
Schematic illustration and DLS readout showing (**i**) Conjugation of ssDNA Seq A and Seq B respectively to AuNPs, exhibiting a single peak/population on DLS (**ii**) ERE-containing AuNP dimer formation, which also showed a single peak a at ~10 nm right-shift compared to the conjugates (**iii**) Addition of ERβ to the as prepared ERE-containing AuNP dimer sensing probes, and the presentation of a two-peak readout with an additional complex peak at around 200 nm. Observations of TEM images showed AuNP size and distributions generally corresponded to the DLS hydrodynamic diameter readouts.

Figure 1 shows the design and assay principle wherein 11 nm AuNPs probes of defined dimeric structures, linked by a ERE-containing DNA duplex are used to investigate the interactions between ER (all mentions of ER refers to the β subtype, unless specifically stated otherwise) and their binding sites. To form the dimer probe, two sets of AuNP conjugates bearing 80 mer single ssDNA (AuNP-ssDNA) of Seq A and Seq B (Supplementary Fig. S1) were first prepared through stoichiometric control (i.e., one DNA strand per AuNP), followed by purification using agarose gel electrophoresis (Supplementary Fig. S2). DLS measurement of these AuNP-ssDNA (Seq A or B) showed a single distinct population with size distribution peak around 20 nm, which correlated well with the TEM images of these conjugates as well-dispersed individual nanoparticles. A 100 mer DNA linker (consisting of two 50-mer sequence complementary to Seq A and Seq B respectively) was designed to bridge the two sets of AuNP-ssDNA conjugates (i.e., AuNP-Seq A and AuNP-Seq B) and formed a double-stranded DNA (dsDNA) bridged dimeric nanostructure construct. This AuNP dimer contained the consensus wildtype ERE sequence (GGTCAnnnTGACC) located at Seq B where ER can recognize and specifically bind to. The ERE-containing AuNP dimer was then purified on agarose gel (Supplementary Fig. S3) and recovered using electrophoretic dialysis. When characterized by DLS measurement, the purified ERE-containing AuNP dimers showed a ~10 nm rightward peak shift relative to that of the individual conjugates (20 nm). The formation of the AuNP dimer construct was further confirmed by TEM images (Fig. 1). The as-formed 30 nm ERE-containing AuNP dimers can then be used as a highly specific sensing probe to detect DNA-ER binding interactions in homogenous solution: The DLS readout showed the appearance of a ‘complex peak’ in the 200–300 nm region, which was accompanied by a decrease in the DLS signal intensity of the original dimeric peak at 30 nm (Fig. 1). These distinctive, two population, optical signature is believed to be the result of the sequence-specific binding of ER onto the ERE-containing AuNP dimers, which marked the first time that such nanostructures is used in conjunction with DLS platform to study transcription factor-DNA interactions.

**Figure 2:**
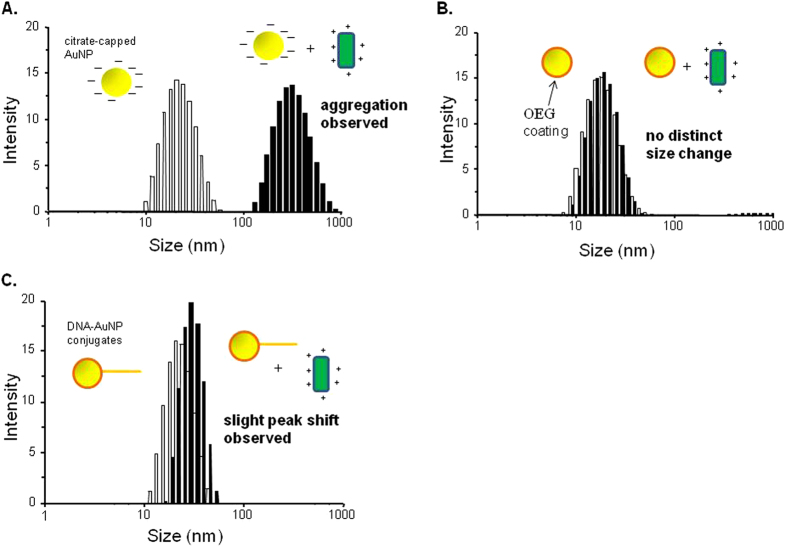
DLS analysis of the ERβ interaction with (**A**) unmodified citrate-anion capped AuNPs (**B**) OEG passivated AuNP (**C**) AuNPs bearing one strand of ssDNA. The particle size distribution of different AuNPs systems before and after addition of 10 nM of ERβ are indicated by the ‘empty’ and ‘solid’ bar charts, respectively.

To better establish the phenomenon of ER and ERE-containing AuNP dimer interaction, DLS analysis of ER interaction with different AuNP nanostructures, namely unmodified citrate-anion capped AuNPs, OEG passivated AuNP, and AuNPs bearing one strand of ssDNA was conducted. First, citrate-anion capped AuNPs (starting material used to fabricate DNA-linked AuNP dimers) was incubated with 10 nM ER. This resulted in significant particle aggregation and shifted the entire population of AuNPs on the DLS readout from 20 nm (empty bar) to around 500 nm (solid bar) as shown in Fig. 2A. When ER interacted electrostatically with AuNPs, the positive charge of the proteins negated the negative charge of the AuNPs, reducing the interparticle repulsion and induced the irreversible, bulk aggregation of the AuNPs^24^. It is also known that basic residues and thiol moieties on proteins can interact with the negative charge of the AuNP surface^25^,^26^. The ER-mediated aggregation of citrate-capped AuNP was also observed in the work of Tan and co-workers on ER detection using AuNPs^19^. Further investigating the effects of surface charge alterations on the colloidal stability of AuNPs, OEG-capped AuNPs, formed from citrate-capped AuNPs passivated with OEG at a 500 fold OEG:AuNP ratio, were also tested with ER. As shown in Fig. 2B, the OEG-capped AuNPs were highly stable in the presence of ER, with the DLS readouts being essentially identical before and after ER addition. AuNPs capped with OEG retained their negative charge status and remained discrete particles, and were passivated against ER and other AuNPs, thus the aggregation previously observed for the citrate-capped AuNPs was not observed. This mechanistic insight was further augmented when single AuNP-ssDNA conjugates was incubated with ER, a slight rightward peak shift of ~10 nm was observed (Fig. 2C), but with no complex peak. This could be explained by the non-specific interaction of ER with the ssDNA attached to the AuNP, which was unable to induce AuNP clustering and cause significant size increase. On the other hand, we have also found that when the ERE-containing dimers were incubated with ERα, another isoform of ER, results similar to that of ERβ was observed with the appearance of a complex peak (Supplementary Fig. S4). It was only when both ER and ERE-containing AuNP dimers were present that a readout with the complex peak could be elicited, followed by the decrease in population of the original dimer peak. We believe that the specific interaction between ER and its binding site ERE was the cause for this unique optical signature, which could in turn be viewed as a definitive readout for the presence of the ER transcription factor.

A point of note is that the ER-ERE interaction could not be studied on their own on DLS without the transduction of the signal readout by AuNPs (Supplementary Fig. S5). The readouts of the ER-only, and ER-bound ERE samples (all without AuNPs) showed no significant difference from that of buffer only. This was likely due to the biomolecular samples possessing small light scattering cross section that were not clearly distinguished from the background. In addition, the results of OEG-passivated AuNPs (Fig. 2B) had shown that the presence of AuNP could suppress the light-scattering signal from the unbound proteins, buffer and other background noises. These factors suggested a highly sensitive and specific DLS readout with the biorecognition transduced by the unique ERE-containing AuNP dimers for the detection of target transcription factor in complicated biological samples such as blood or cell lysates that have less distinct light scattering cross section.

### Real time detection and concentration dependence of ERE-containing AuNP dimer-DLS readout for ERβ binding

**Figure 3:**
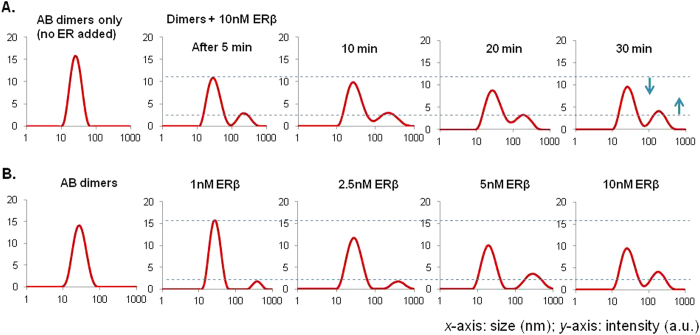
(**A**) Time-dependent study of the ERE-containing AuNP dimers interaction with 10 nM ERβ over 30 minutes. The complex peak was observed at 5 minutes and the decrease in dimer peak intensity was accompanied by the growth of the complex peak (**B**) The dimers were queried with different concentrations of ERβ (1, 2.5, 5 and 10 nM) at 30 minutes after ERβ addition. The intensity of the complex peak relative to the dimer peak was incrementally higher as more ERβ was used.

For bioassay development, it is important to quantify the amount of analytes at low detection limit, as well as to establish the rapidity of the technique. We had first analyzed the changes in the complex peak relative to the original dimer peaks over time upon adding 10 nM ER to the ERE-containing AuNP dimers. As seen in Fig. 3A, the detection took only 5 minutes with the appearance of the complex peak, which suggested that DLS could promptly pick up and visualize any interaction between ER and ERE, mediated by the AuNP dimer probes. Over time, the size of complex peak relative to the dimer peak increased, which could be due to the temporal effects of ER binding onto ERE-containing AuNP dimers. Such time-dependent kinetics was typical of binding processes of DNA-protein interactions, though more remains to be investigated to fully establish this. Furthermore, we demonstrated that the unique optical changes of the complex peak versus the original dimer peak was ER concentration-dependent. Figure 3B showed the particle size distribution of ERE-containing AuNP dimers in the presence of different amounts of ER. Larger size changes was observed to correspond to greater amounts of ER added (at 30 minutes after ER addition). The results suggested the amount of proteins could also be quantified. Potentially, the sensitivity of the technique could be enhanced through further optimizations in the amount of probes used and the binding conditions to maximize ER binding to ERE-containing AuNP dimers. One distinct advantage of DLS over conventional spectroscopic technique as determined from our works was that the amount of probes required to elicit the readout was much lesser as a high threshold concentration of AuNP was not required, unlike colorimetric or UV spectroscopy-based detection techniques^27^,^28^, while at no discount of the speed and ease of readout.

### Sequence and target selectivity of the ERβ binding detection system

**Figure 4:**
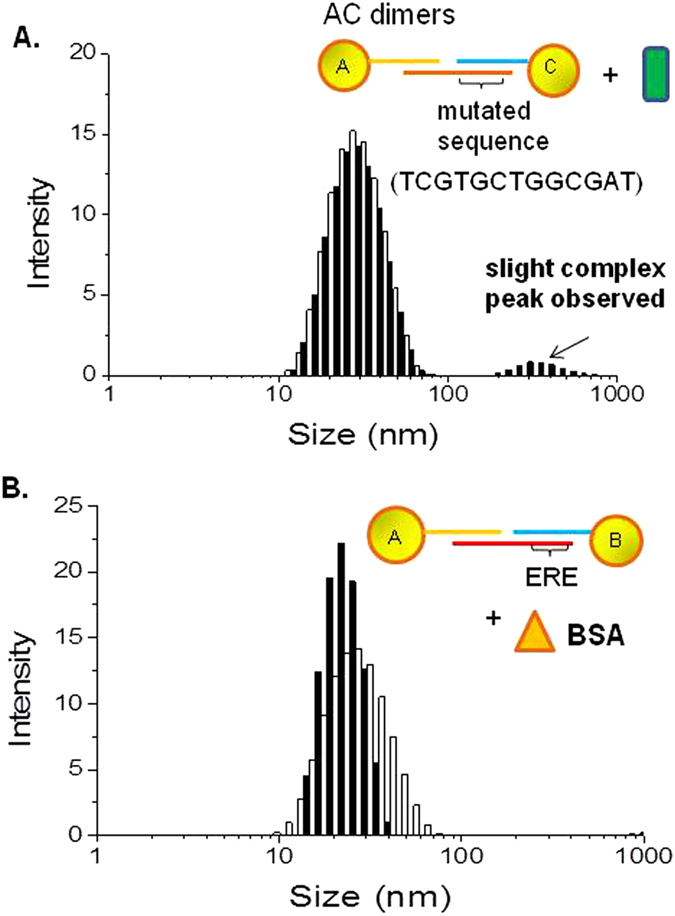
(**A**) AC Dimers consisting of 11 nm AuNPs joined by dsDNA containing mutated ERE sequence was fabricated and tested with 10 nM ERβ. Complex peak was observed, but at a low intensity relative to the dimer peak (**B**) ERE-containing AuNP dimers were queried with a non-specific protein – BSA. No complex peak was observed. All results were taken at 30 minutes after incubation with protein.

To highlight the sequence selectivity of the technique, a different type of dimers AC, was fabricated from Seq A and Seq C AuNPs conjugates carrying one ssDNA and linked by a 100-mer AC linker. Different from the ERE-containing AuNP dimers, the AC dimers contained a mutated DNA sequence located at the Seq C where the core binding sequence of ERE was scrambled. When 10 nM of ER was added to the AC dimers probes for DLS measurement, a complex peak was observed, but at a much lower intensity as compared to their ERE-containing counterparts (Fig. 4A). This could be attributed to the electrostatic interactions between the AC dimers and ER. However, due to non-specific and weak binding nature of the ER-AC dimer interactions, the ensuing complex peak was at a much lower intensity, relative to that observed for ERE-containing AuNP dimers.

To establish the system specificity for the target protein, the same ERE-containing AuNP dimers were queried with bovine serum albumin (BSA). At comparable concentrations of protein, we found that the size of the system was essentially unchanged, and that no complex peaks were observed (Fig. 4B). Since changes in the transcription factors levels in cells are the subject of much scientific curiosity, such as the reprogramming of stem cells and study of oncogenic pathways^29^,^30^, any system querying the cell extract has to be minimally affected by the presence of many different proteins and not give any non-specific readouts. In this case, an unrelated protein (BSA) was unable to elicit any aggregation in ERE-containing AuNP. While proteins are known to induce AuNP aggregation through charge interaction, the OEG passivation of the AuNPs would prevent this from happening, thus maintaining the specificity of the DNA-bridged dimers for the ER target.

## Discussion

AuNPs and DLS are two highly complementary platforms as the large scattering cross section of the AuNPs facilitates a clear and distinct DLS readout^17^. Given further that the DLS signal is amplified with larger AuNPs, and increases in the system size further enhance the signal readout, there is motivation to combine both AuNPs and DLS in a detection system. Our system was designed such that ERE-containing AuNP dimer could interact with ER through specific binding of the protein that eventually presented as an unique DLS readout: 1) The localization of positively-charged ER on the ERE negated the negative charge of the AuNPs, and 2) The reduction of the electrostatic repulsion provided a driving force for their clustering, 3) AuNPs in a dimer exerted steric hindrance towards the binding of ER on ERE, such that only specific interactions would be favoured. In addition, as ERβ binds to ERE as a tetramer, a few ER-bound AuNP dimers would cluster as their respective ERs are assembled or interacted non-specifically through the protein side chains^31^. Moreover, ER binding resulted in the distortion of the response element with bending towards its major groove^32^, which inadvertently caused the AuNPs in a dimer construct to come into closer proximity. Such plasmonic coupling would also contribute to the red shift and increase in light scattering signature^33^,^34^, which enhanced the signal readout. All these led to the increase in the overall size of the system and amplified the intensity of the DLS readout. Comparing to the 50 nM detection limit of ER reported by Tan and co-workers using an AuNP-based colorimetric assay, the AuNP-DLS tandem we show is at least 10 times more sensitive^19^.

The design and mechanism of our detection system is different from typical AuNP-DLS systems for protein detection based on aggregation of AuNPs for enhancement of DLS readout. Usually, aggregation-based systems requires probes that are saturated with DNA or antibody, and their readouts typically show nm-to-micron transitions which can vary widely^14^,^22^. AuNPs aggregate when they lose their colloidal stability, which can be attributed to electrostatic and steric factors, and environmental conditions as the presence of ions like Na^+^ and Cl^–^ can negate the AuNP surface charges, and such screening effect leads to increased clustering and aggregation. Thus, it is imperative to maintain the Coulombic repulsion and ensured the colloidal stability of the system until the target is introduced^35^,^36^. The unique design with ERE localized in the OEG-passivated dimers imparted a level of control such that only specific dimer-protein interaction could induce a change in the colloidal stability of the system. The presence of an additional AuNP in a dimeric construct not only increases the scattering cross section, but also exerts a steric effect against non-specific binding. As a result AuNPs would cluster to certain extent of particle stability instead of aggregating uncontrollably, leading the observed readout of the decrease in dimer peak intensity accompanied by the appearance of a complex peak when ER was added to ERE-containing AuNP dimer. This stability also ensured that readout changes, if any, must be due to the presence of the protein target. In addition, our work is distinct from techniques based on the use of single AuNPs for protein detection. As reported by Su and co-workers, the DLS size of a multiple dsDNA-AuNPs monomer only increase from 32 nm (DNA saturated 13 nm AuNPs) to 53 nm (after protein binding)^37^. The saturation of dsDNA on the AuNPs surface prevented multiple proteins from binding effectively to DNA-AuNPs conjugates due to steric effects. And even if the dsDNA density allows proteins to bind effectively, no significant size increase is expected in the monomer sensor due to the limited size of the protein (a few nm). Hence appreciable size increase was not observed on DLS.

**Figure 5:**
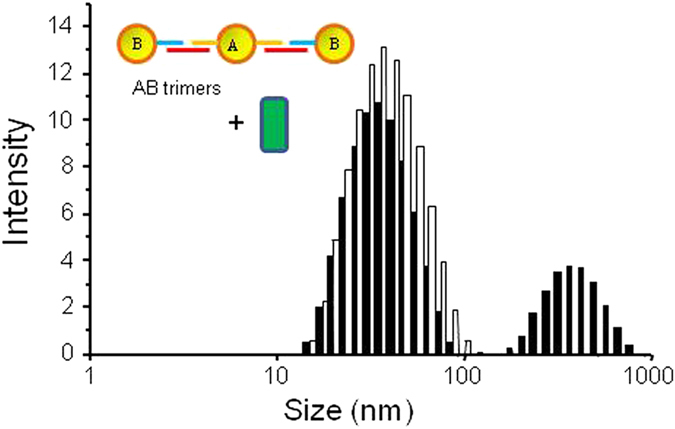
DLS readouts ERE-containing trimers incubated with ERβ. Complex peak was observed, centered around 300–400 nm size range.

In effort to improve the detection process, the design of our system was evolved with the fabrication of ERE-containing AuNP trimers consisting of a central AuNP carrying two Seq A, which were bound to respective AuNP conjugates carrying Seq B. This also created two binding sites for ER, and has the potential for more extensive interaction between higher order AuNP nanostructure and ER. From Fig. 5, it was clear that upon incubation with ER, the ERE-containing AuNP trimers showed a readout with two peaks, with complex peak once again observed. On the one hand, this validated the design of the system in which AuNP nanostructures are utilized for transcription factor detection; On the other hand, while the complex peak is located at a larger size average than that observed for the dimers, the readout was not significantly different. The presence of the added AuNP on the trimeric nanostructure could exert a steric effect, and prevent the approach of ER in binding to ERE. Also, the trimer is not in a perfect linear form and the bridging DNA could twist and bend. As a result, the steric effect would become more pronounced, which modulated the detection outcome in spite of the electrostatic effects due to greater probe-protein interactions.

The use of nanostructures with a large scattering cross section also reduced the amount of AuNP probes required to bring about meaningful signal changes. In fact, AuNPs samples that showed no appreciable signal on the UV-*vis* spectroscopy would still present a clear signal on DLS measurement (Supplementary Fig. S6), which is an additional advantage of using DLS for bimolecular detection over the conventional spectroscopic techniques. Thus, unlike conventional AuNP detection systems where the AuNP probes are used excessively, the amount of probes used here could be purposefully kept low such that even if ER is at low concentrations, their interaction with ERE could still elicit an appreciable positive readout. Last but not least, this is a label-free detection method where ER is detected in its native form, as desired in biomarker sensing in general.

In conclusion, we present a novel ERE-containing AuNP dimer probe that is used for the detection of ER protein, via the signature readout on DLS. Complex peaks were observed only in the presence of ERE and ER, thus showing sequence specificity and protein selectivity. The quantification potential of the system was also demonstrated through protein concentration dependent DLS signal output. Moreover, the system could provide a readout as quickly as 5 minutes, which puts it in good stead as a bioassay system for the study of not just transcription factors, but also other valuable biomarkers. This assay provide a low nM level sensitivity which is more favorable than other AuNP-based aggregation assays that measure bulk-phase changes of particle size under UV-*vis* spectroscopy. Not just limited to ER protein detection, this single-tube ‘mix and test’ AuNP dimer DLS-based bioassays offer flexibility for detecting other DNA binding molecules by simply changing the conjugated DNA sequence, making them versatile probes for use in biomedical research and diagnostic applications.

## Methods

### Materials

Tetrachloroauric (III) acid, *4,4′* (phenylphosphinidene) bis(benzenesulfonic acid), dipotassium salt hydrate 97% (PPBS) and thiolated methoxy-hexa(ethylene glycol) (OEG) were purchased from Sigma-Aldrich. All oligonucleotides were purchased from Integrated DNA Technologies. ER was purchased from Life Technologies.

### Synthesis and characterization of gold nanoparticles

11 nm AuNP synthesis via the reduction of HAuCl_4_.4H_2_O using citric acid and tannic acid was as described by Handley *et al.*^38^. The citrate-capped AuNP were characterized first with UV-*vis* spectroscopy (Varian Cary 50Bio) and DLS (Zetasizer Nano ZS, Malvern), followed by TEM (JEOL JEM-2100F, 200 kV). The electron micrographs were analyzed with the Image J software (NIH), over an average of at least 200 particles. The standard deviation of the as-synthesized AuNPs was consistently within 10% of the average size.

### Fabrication and recovery of dimers

The 11 nm AuNP synthesized were first passivated with 10 mg/ml PPBS in a 50:1 AuNP to PPBS volume ratio for at least 4 hours, which stabilized the AuNPs against aggregation in the salt conditions used for conjugation^39^. This was followed by incubation with Seq A, Seq B or Seq C, and then passivation with thiolated OEG. These AuNP-ssDNA conjugates were raised in an environment of 200 mM NaCl, to which 5 M NaCl was added the day after to gradually raise the salt conditions to 600 mM. The conjugates were subsequently washed in 50 mM Tris and NaCl buffer. Conjugates were ran on a 3% agarose gel (5 V/cm, 180 min) and conjugate monomers bearing single ssDNA were identified based on their relative electrophoretic mobilities and recovered through excision of relevant gel area, followed by electrophoretic dialysis and concentration. The concentrations of the probes were estimated using UV-*vis* spectrometry, based on the reported absorption coefficient parameters^40^. For the formation of dimers, A and B conjugate monomers were hybridized to their complementary target (AB), and A and C conjugate monomers to target AC, with the target amount fixed at 1 pmol and at a 1:1 probe to target ratio. Hybridization conditions of 100 mM Na^+^ and 2 mM Mg^+^ were used as reported^41^. The hybridized samples were left to stand for at least an hour. After which, a 3% agarose gel at 75 V for 45 minutes under room conditions was used to actualize the formation of defined AuNP dimers. AuNP dimers were subsequently recovered through the excision and electrophoretic dialysis of the dimer gels. The recovery of the desired dimeric nanostructures was determined by TEM and DLS as well as further gel runs, ensuring that dimers were successfully isolated and remained stable. Dimer concentration was estimated from UV-*vis* spectroscopy and based on parameters reported in literature^40^.

For AuNP trimer construction, the DNA to AuNP stoichiometry used in the conjugation process was increased to obtain AuNP-Seq A with 2 ssDNAs as the major product (as the densest band). Purified AuNP-Seq A with 2 ssDNA attached was incubated with AuNP-Seq B carrying single ssDNA and linkers, in a 1:2:2 ratio, to promote the formation of AuNP trimers. The hybridized samples were purified on agarose gel, and recovered by electrophoretic dialysis, followed by TEM and DLS characterizations.

### Incubation of dimers with ERβ protein and test on dynamic light scattering platform

ERβ was stored at −80 °C and, once thawed, was added immediately into a cuvette containing the dimers characterized just prior to the experiment in 50 mM Tris + NaCl buffer. The protein and dimers were mixed well through gentle pipetting for ER binding while the dimers remained stable. The cuvette was then placed into the holder in the zetasizer, and the content was allowed to incubate while the machine equilibrated and optimized the detection settings. Each measurement run was over 10 seconds and an average of about 15 measurements was done per sample. For temporal studies, the AuNP dimers-ER mixture was kept under gentle shaking, with measurement taken at the appropriate time point.

## Additional Information

**How to cite this article**: Seow, N. *et al.* DNA-Directed Assembly of Nanogold Dimers: A Unique Dynamic Light Scattering Sensing Probe for Transcription Factor Detection. *Sci. Rep.* **5**, 18293; doi: 10.1038/srep18293 (2015).

## Supplementary Material

Supplementary Information
