# Supplementary material for: DNA-Directed Assembly of Nanogold Dimers: A Unique Dynamic Light Scattering Sensing Probe for Transcription Factor Detection
Source: Sci Rep. 2015 Dec 18;5:18293. doi: 10.1038/srep18293 (PMC4683372; doi:10.1038/srep18293)
Supplement: Supplementary Information [file srep18293-s1.pdf]

DNA-Directed Assembly of Nanogold Dimers: A Unique Dynamic Light Scattering Sensing Probe for Transcription Factor Detection

(Supplementary Information)

Nianjia Seow[a], Yen Nee Tan[b*], Lin-Yue Lanry Yung[a*], Xiaodi Su[b]

[a]Department of Chemical and Biomolecular Engineering, Faculty of Engineering, National University of Singapore, Singapore 119260, Singapore

[b]Institute of Material Research and Engineering, ASTAR (Agency for Science, Technology and Research), 3 Research Link, Singapore 117602

[*]L.L.Y.: cheyly@nus.edu.sg ; Y.N.T.: tanyn@imre.a-star.edu.sg

Seq A (80b):
5' (SH) –TTT TTT TTT TGG ACA TCA AAA TGT GTT TTT TAC TGG GAC TTT TGT TCA TAA AAT AGT TTT CTG CAT AGC CTC TTT GATTA

Seq B (80b):
5' GAT TAA CTG TCC AAA GTC AGG TCA CAG TGA CCT GAT CAA AGT TAA TGT AAC CTC AAC CTG GAC AAA GCT ACT AGT TTT TT– (SH) 3'

Seq C (80b):
5' GAT TAA CTG TCC AAA GTC AAT CGC CAG CAC GAT GAT CAA AGT TAA TGT AAC CTC AAC CTG GAC AAA GCT ACT AGT TTT TT –(SH) 3'

Complementary target A-B (100b):
5' TTACATTAACTTTGATCAGGTCACTGTGACCTGACTTTGGACAGTTAATCTAATCAAAGAGGCTATGCAGAAAACTATTTTATGAACAAAAGTCCCAGTA

(Consensus sequence for ER binding underlined)

Complementary target A-C (100b):
5' TTACATTAACTTTGATCATCGTGCTGGCGATTGACTTTGGACAGTTAATCTAATCAAAGAGGCTATGCAGAAAACTATTTTATGAACAAAAGTCCCAGTA

Supplementary Figure S1. DNA sequence of Seq A, SeqB, Seq C, complementary target AB and complementary target AC. AuNP conjugates carrying Seq A and Seq B form ERE-containing AuNP dimers in the presence of the AB linker; Conjugates A and C form AC dimers in the presence of target AC.

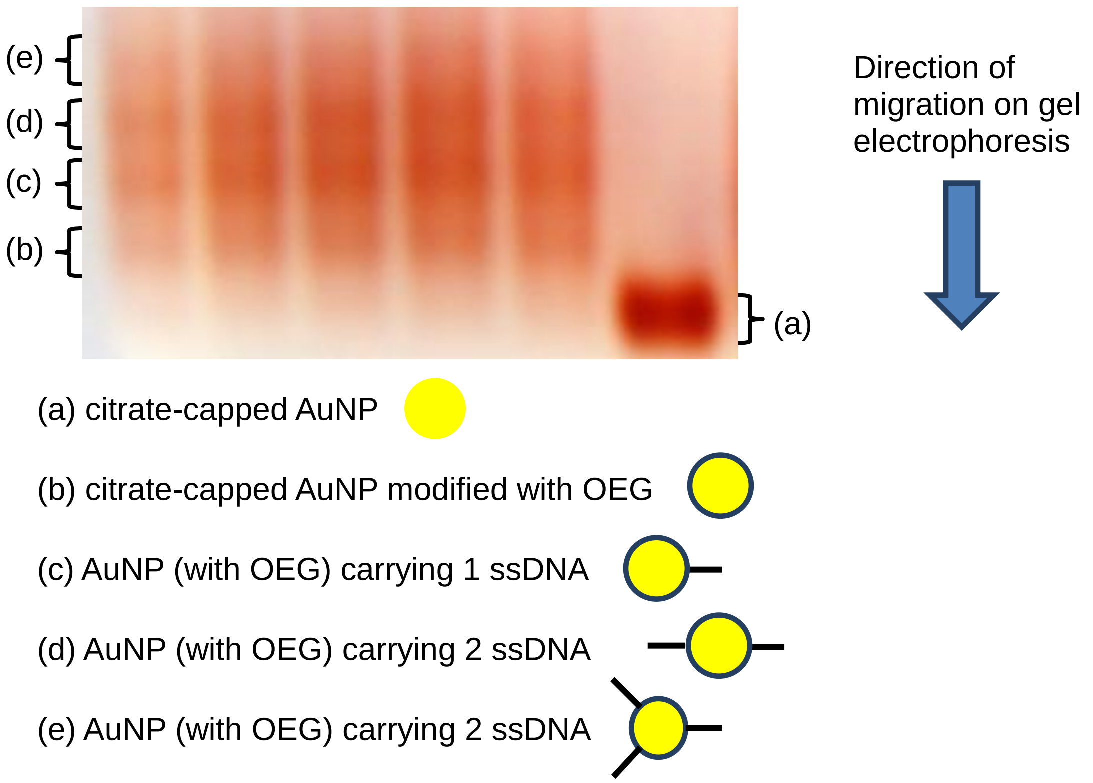

Supplementary Figure S2. 11nm AuNPs functionalized with 80b ssDNA results in distinct bands on the agarose gel corresponding to the number of ssDNA conjugated.

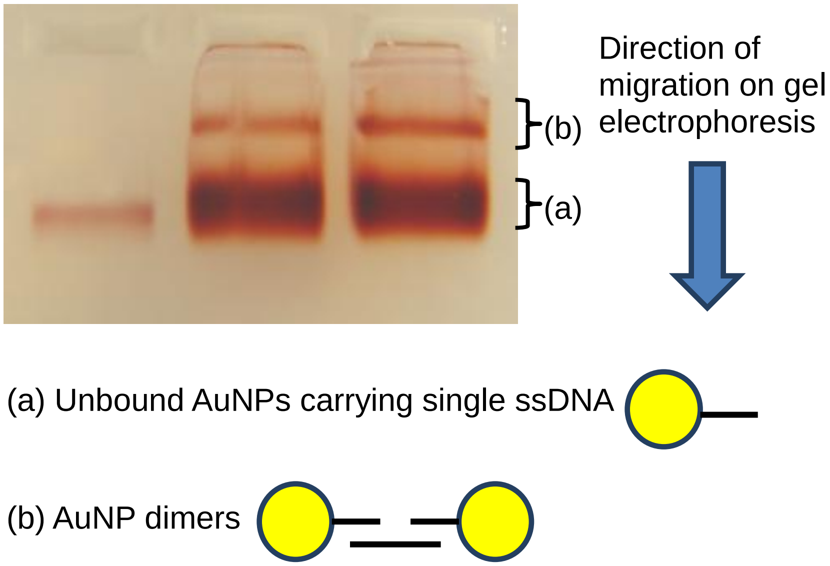

Supplementary Figure S3. Conjugates bearing single ssDNA were recovered and purified and hybridized to a 100b linker, which resulted in the formation of dimeric nanostructures. These dimers were then used to detect ER.

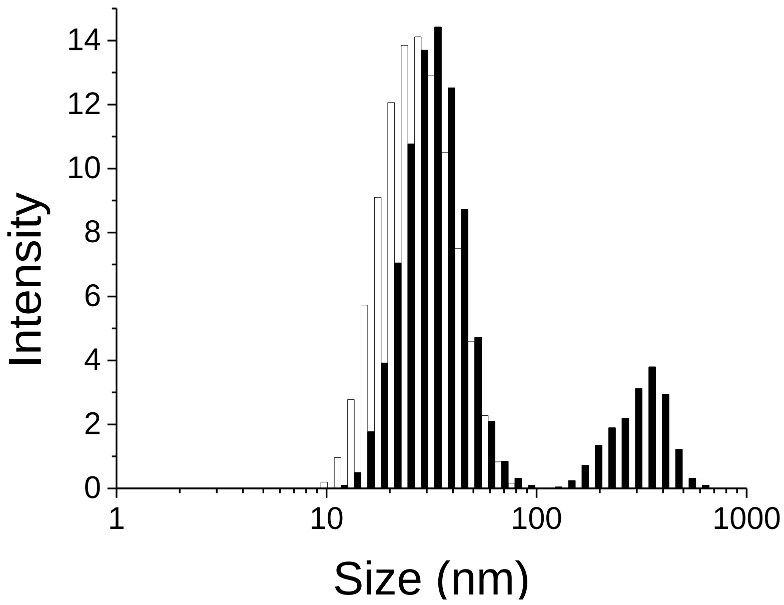

Supplementary Figure S4. DLS readout of ERE-containing AuNP dimers with 2.5nM ERα.

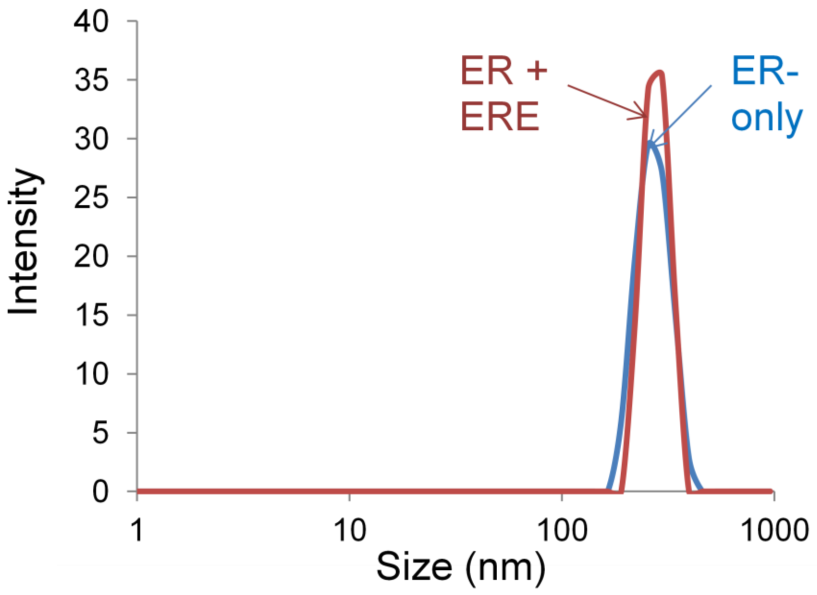

Supplementary Figure S5. DLS readouts of ER-only and ER+ERE systems, in the absence of AuNPs.

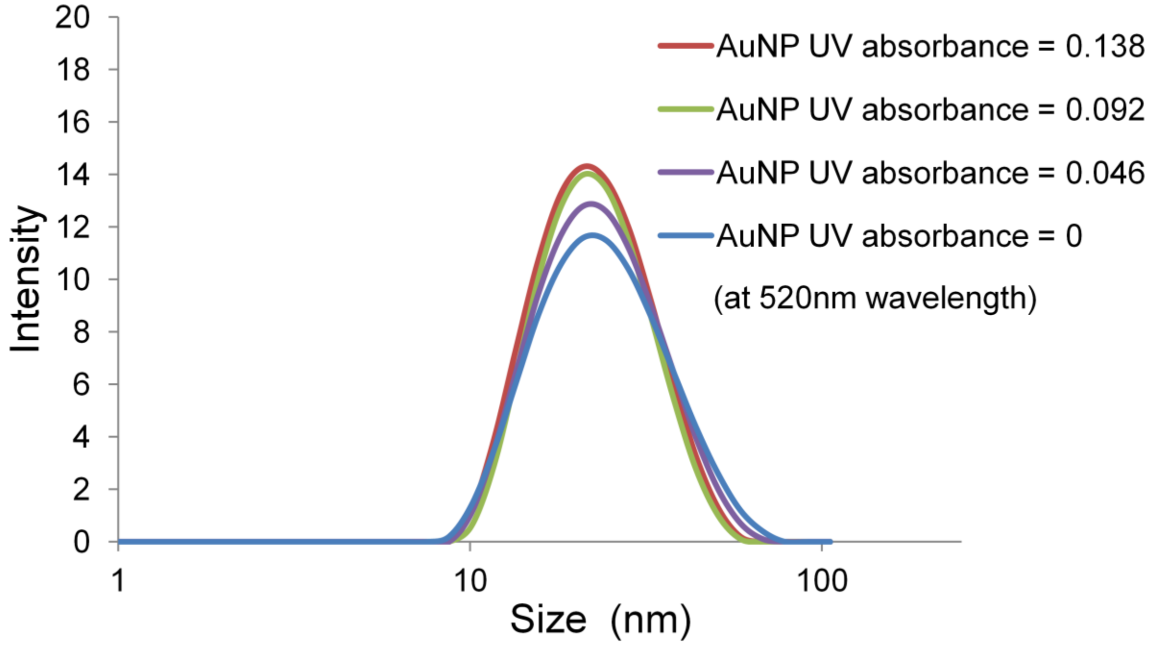

Supplementary Figure S6. DLS readout of 4 different samples of unmodified AuNP, showing different UV absorption values.
